# MAGAT gel and EBT2 film‐based dosimetry for evaluating source plugging‐based treatment plan in Gamma Knife stereotactic radiosurgery

**DOI:** 10.1120/jacmp.v13i6.3877

**Published:** 2012-11-08

**Authors:** N. Gopishankar, S. Vivekanandhan, S.S. Kale, G.K. Rath, S. Senthilkumaran, S. Thulkar, V. Subramani, M.A. Laviraj, R.K. Bisht, A.K. Mahapatra

**Affiliations:** ^1^ Gamma Knife Unit Department of Neurosurgery Neurosciences Centre All India Institute of Medical Sciences Ansari Nagar New Delhi India; ^2^ Department of Neurobiochemistry Neurosciences Centre All India Institute of Medical Sciences Ansari Nagar New Delhi India; ^3^ Department of Radiation Oncology Dr. B. R. Ambedkar Institute Rotary Cancer Hospital All India Institute of Medical Sciences Ansari Nagar New Delhi India; ^4^ Department of Nuclear Magnetic Resonance All India Institute of Medical Sciences Ansari Nagar New Delhi India; ^5^ Department of Radio Diagnosis All India Institute of Medical Sciences Ansari Nagar New Delhi India

**Keywords:** Gamma Knife, MAGAT Gel, EBT2 film, source plugging, 3D dose distribution

## Abstract

This work illustrates a procedure to assess the overall accuracy associated with Gamma Knife treatment planning using plugging. The main role of source plugging or blocking is to create dose falloff in the junction between a target and a critical structure. We report the use of MAGAT gel dosimeter for verification of an experimental treatment plan based on plugging. The polymer gel contained in a head‐sized glass container simulated all major aspects of the treatment process of Gamma Knife radiosurgery. The 3D dose distribution recorded in the gel dosimeter was read using a 1.5T MRI scanner. Scanning protocol was: CPMG pulse sequence with 8 equidistant echoes, TR=7 s, echo step=14 ms, pixel size=0.5 mm x 0.5 mm, and slice thickness of 2 mm. Using a calibration relationship between absorbed dose and spin‐spin relaxation rate (R2), we converted R2 images to dose images. Volumetric dose comparison between treatment planning system (TPS) and gel measurement was accomplished using an in‐house MATLAB‐based program. The isodose overlay of the measured and computed dose distribution on axial planes was in close agreement. Gamma index analysis of 3D data showed more than 94% voxel pass rate for different tolerance criteria of 3%/2 mm, 3%/1 mm and 2%/2 mm. Film dosimetry with GAFCHROMIC EBT 2 film was also performed to compare the results with the calculated TPS dose. Gamma index analysis of film measurement for the same tolerance criteria used for gel measurement evaluation showed more than 95% voxel pass rate. Verification of gamma plan calculated dose on account of shield is not part of acceptance testing of Leksell Gamma Knife (LGK). Through this study we accomplished a volumetric comparison of dose distributions measured with a polymer gel dosimeter and Leksell GammaPlan (LGP) calculations for plans using plugging. We propose gel dosimeter as a quality assurance (QA) tool for verification of plug‐based planning.

PACS number: 87.53.Ly, 87.55.‐x, 87.56.N‐

## I. INTRODUCTION

Over the past five decades, LGK (Elekta Instrument AB, Stockholm, Sweden) has become the standard of care for stereotactic radiosurgery (SRS). Gamma Knife Model B consists of 201 cobalt‐60 sources that emit gamma ray photons that allow submillimeter accuracy delivery of radiation to the specified area without damaging surrounding brain tissue. Single or multiple isocenters (shots) using the four available helmets (4 mm, 8 mm, 14 mm, 18 mm) can be used to treat targets with a wide variety of shapes. The dose is usually prescribed to the periphery at 50% isodose level, although higher prescription isodoses up to 80% may also be used in some cases. It is of vital importance that the radiological characteristics of the treatment unit of a radiosurgical system be verified regularly. The radiosurgical targets are generally surrounded by delicate neurological structures which may be easily damaged. Radiation dose to the critical structures in the vicinity of the clinical target is limited by blocking radiation entering the target through the critical structures using shields called plugs (made of 96% tungsten alloy and 6 cm length). This form of placement of shield is called plugging in Gamma Knife treatment planning, and it modifies the dose distribution and aids in dose reduction.

Gamma Knife plans with plugging are optimized using a shielding feature in the LGP software that generates a plug pattern on the structures at risk while creating a radiosurgical plan. Dose calculations of LGP are based on interpolated empirical data. Superposition technique of radiation distributions from all gamma beams is used to calculate dose in the patientís head. The dose contribution by each individual beam relies on the inverse square law, exponential attenuation using the linear attenuation coefficient and single beam profiles.^(1)^ In a plug plan, the plugged beams are excluded from the overall dose calculations and hence the plug modifies the radiation distribution. The LGP system (LGP Version 8.0) does not take into account tissue heterogeneities, leading to larger dose uncertainty if the treatment volume is near an air–tissue interface or the skull surface.^2^, ^3^ Additionally, the external head contour is defined using 33 discrete points, leading to possible uncertainties for patients with irregular skull shapes.

All treatment planning systems contain uncertainties, all of which can affect the accuracy with which planning and treatment are done. One of the sources of uncertainties is in dose calculation.^(4)^ The QA program recommendations on SRS by Hartmann^(5)^ emphasize that computerized treatment planning needs to be verified by measurement. The Task Group 42 Report does recommend measuring beam profiles with all but one collimator plugged, but verification of TPS calculations for plugging is not specifically addressed in that report.^(6)^ Plan‐specific quality assurance testing has historically been considered of less importance due to the robustness of the Gamma Knife system and the extensive experience of the Radiation Oncology community in this treatment technique.

Researchers, however, have shown interest in designing their own QA strategies for verification purpose in Gamma Knife radiosurgery. Previously, numerous studies were done to analyze the accuracy of plugging in Gamma Knife.^7^, ^8^, ^9^, ^10^ There are a set of Monte Carlo (MC) studies which were successfully used for simulating Gamma Knife sources and units itself, including plugging patterns.^2^, ^7^, ^10^, ^11^, ^12^, ^13^, ^14^, ^15^ Cheung et al.^(7)^ employed Monte Carlo general‐purpose code EGS4 implementing PRESTA (parameter reduced electron‐step transport algorithm) algorithm and GAFCHROMIC films (Type MD 55)^16^, ^17^ to verify the accuracy of LGP TPS when plugged collimators were used.

In the past, MC techniques were considered to consume great amounts of computing resources, too much to be of practical use for patient‐specific QA.^(18)^ However in recent times, MC simulation of radiation transport in patient geometry with the high‐performance computing (parallel processing), or a multicore processor workstation, can bring the results for the GK plan verification within one hour. On the other hand, film measurements for GK verification give accurate measurements, but are restricted to planar measurements.^(19)^

In recent times, 3D dosimetry, such as gel dosimetry, has gained popularity because it has the potential to measure highly complex treatments, which is particularly significant in situations where steep dose gradients exist such as intensity‐modulated radiation therapy (IMRT) and SRS.^20^, ^21^, ^22^, ^23^, ^24^, ^25^, ^26^ This 3D measurement method simulates all major aspects of the treatment process, and provides 3D dose distributions measurements with high resolution.

MAGIC gel was used for 3D dose verification in radiosurgery previously by several authors.^3^, ^27^, ^28^ MAGAT gel, which is a modified version of MAGIC gel, was not tested for radiosurgery verification. Brindha et al. and Hurley et al.^29^, ^30^ show in their studies that a MAGAT formulation with 10 mM of THPC, 0 mM HQ, 8% gelatin, and 9% methacrylic acid was found to give the greatest change in spin‐spin relaxation rate (R2) with dose and, for this reason, this type of gel was used for the present work. A 9% methacrylic‐based gel was found to give linear doses response up to 40 Gy and is ideal for performing radiosurgery verification.^(31)^ Several studies exist for Gamma Knife verification with polymer gel dosimetry.^3^, ^13^, ^31^, ^32^, ^33^, ^34^, ^35^, ^36^ Interestingly, 3D measurement based on gel dosimetry has not been used so far for plug planning verification which is the goal of this study.

## II. MATERIALS AND METHODS

### A. Gel preparation

The gel dosimeters used in this experiment were normoxic “MAGAT” gels which were prepared in‐house at normal atmospheric conditions. A volume of 3000 ml of gel was prepared using 9% methacrylic acid, 8% gelatin, 83% triple deionized water, and 10 mM THPC.^(28)^ After manufacture, the polymer gels were poured into a 16 cm diameter spherical glass phantom mimicking the human head and small glass tubes for calibration, and stored at 5°C overnight in a refrigerator.

### B. Preirradiation scanning

The spherical phantom was fixed in the Leksell stereotactic frame using a set of four vinyl suction cups. In this study instead of metallic posts, carbon material posts (Elekta AB, Stockholm, Sweden) were used in order to avoid any artifacts during image acquisition. After frame fixation, the phantom was left in the MRI scanning room for approximately 24 hrs before scanning, to attain thermal equilibrium. A MR imaging session of a gel phantom stereotactic frame was performed on a 1.5 T Siemens Sonata MR imager (Siemens AG, Erlangen, Germany) with a head coil using T1‐MPR sequence with TR=2320 ms, TE=4.38 ms, FOV=256×256, pixel size $=0.5\times 0.5\;{\text{mm}}^{2}$, NEX=1 (all identical to the ones used for Gamma Knife patient imaging).

### C. Treatment planning and irradiation

**Figure 1:**
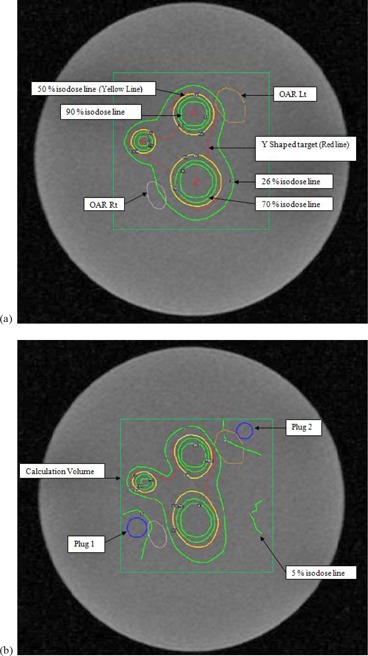
Treatment planning created in LGP with MR images of the MAGAT gel‐filled phantom: (a) plan created with three shots without plugging; (b) plan modified by placement of plugs near the shots represented by small circles in blue color.

The obtained images were imported into the LGP‐TPS for generating a treatment plan. A Y‐shape target (red color) was created for this experiment, as shown in (Fig. 1a). Two organs at risk (OARs) were created on either side of the target to demonstrate the effect of plugging. A Y‐shaped target with the two OARs on either side of the target resembles a pituitary adenoma tumor, which has optic nerves on either side. Three shots were planned with 18 mm, 14 mm, and 8 mm collimator. A grid size of 2.5 mm was set for dose calculation. The prescription dose was 8 Gy to 50% isodose level. After placement of shots, a plug pattern was generated around the shots (shown as two blue rings) near the OARs to create shielding (Table 1). A plug pattern presented in (Fig. 1b) was automatically generated by LGP. Each shot had its own plugging positions in the collimator helmet. The phantom along with the stereotactic frame was mounted on the LGK model B, and the plan was treated.

**Table 1:** Summary of the dose plan created for irradiation in spherical glass phantom.

*Shot. No*	*X (mm)*	*Y (mm)*	*Z (mm)*	*Gamma Angle (deg)*	*Plug Mode*	*No. of Plugs Used*	*Collimator (mm)*
1	102	84	103	100	Auto	45	18 mm
2	101	110	101	100	Auto	21	14 mm
3	76	95.5	106.5	100	Auto	46	8 mm

### D. Calibration

Calibration of the gel was carried out with a Cobalt unit (Theratron 780; AECL, Ottawa, Canada) using SAD technique. The cylindrical glass vials had a wall thickness of 1 mm, an outer diameter of 13 mm, and a length of 95 mm. The vials were placed in the water‐filled tank and irradiated at a depth of 10 cm from the water surface to the middle of the test tube with a single field of 10 cm x 10 cm. To obtain calibration curves, 11 test tubes were irradiated with doses in the range of 0–17 Gy on the same day of treatment planning execution to the spherical glass phantom.

### E. Polymer gel dosimeter evaluation

The irradiated gels were scanned with a 1.5 Tesla Siemens Sonata MR imager using a slice selective 8‐echo Carr‐Purcell‐Meiboom‐Gill sequence with an initial echo time of 14 ms with further increments of 14 ms and a repetition time of 7 s. The slice thickness was selected to be 2 mm without gap between slices. Field of view=256 mm, matrix size=512×512, and pixel size $=0.5\times 0.5\;{\text{mm}}^{2}$. The calibration glass tubes were scanned together with the head phantom. Due to unavailability of MR scanner, the gel dosimeter was scanned after four days. Also considering the access time constraints of MRI, the CPMG spin echo sequence was restricted to 8 spin echoes.

**Figure 2:**
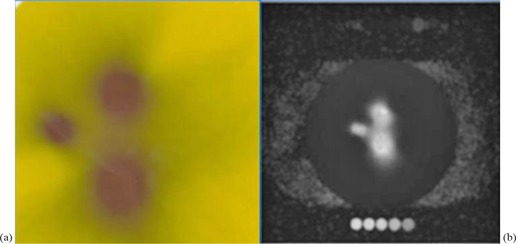
EBT2 film exposed to radiation pattern (a) according to the plan created for plugging. Gel exposure to the same plan (b) seen in the MR image of the gel phantom. Small circles above and below the phantom represent the calibration vials.

The R2 values were computed for the images by assuming an exponential decay of the MR signal using an in‐house MATLAB program (The MathWorks, Natick, MA). A calibration relationship between R2 and absorbed dose was established using the images of the glass tubes (see (Fig. 2b). Then the R2 images of the glass sphere that was treated in the Gamma Knife unit were converted to dose images by applying the calibration equation.^37^, ^38^, ^39^ Absolute dose distribution measurement was not evaluated to avoid introducing errors to the measurement due to the difference in the response of gel vials of different volumes, since the calibration vials were much smaller than the spherical phantom. Measure dose values were scaled by multiplying the original gel dose with a factor of 0.85. This dose scaling factor was found by comparing the dose measured by the MAGAT gel using the calibration data with the dose calculated by LGP in a relatively uniform region inside the treatment volume.^(40)^

**Figure 3:**
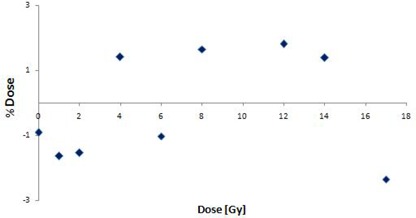
Residual plot for the R2 dose response of the MAGAT polymer gel dosimeter.

For the comparison of experimental data derived with corresponding TPS calculations, the raw data (TPS‐calculated) in a 3D grid suitable for comparison with the 3D matrix of experimental relative dose data were extracted from the TPS (using DICOM RT export function) and imported into MATLAB. Using measured and calculated dose matrices, dose differences at all points in the dose matrix were calculated. The dose difference was defined as a subtraction of the calculated dose from the measured dose.^(41)^ The dose difference values were grouped based on the dose difference and the dose level of the LGP calculation at that point. Three‐dimensional dose comparisons between calculated and measured dose were made by plotting isodose overlay, dose differences, or the gamma evaluation on planes. To analyze the differences in measured and calculated dose in more detail, gamma index analysis was done on a subspace of the measurement volume.^(42)^ For this analysis we choose a paralleopiped subvolume, which included the region around the three shots positioned between X=70 mm to 115 mm, Y=65 mm to 125 mm, andZ=90 mm to 114 mm (see Fig. 3). As the tolerances for the gamma evaluation varying spatial tolerance (0.5–3 mm) and dosimetric tolerance (0.5%–3%) were chosen.

### F. Film dosimetry

Film dosimetry was performed for the same treatment plan and was compared with the calculated TPS dose. For the experimental setup, the spherical polysterene phantom was fixed in the Leksell frame with the four metallic posts and pins. A film insert was placed in the phantom prior to scanning. The phantom was scanned in Philips Big Bore Brilliance X‐Ray CT (Philips Healthcare, Andover, MA). A CT indicator box was fixed in the frame to obtain fiducial markers which was used for planning purpose. Scanning parameters used were 120 kV, 300 mAs, and 2 mm slice thickness. The scanned images were transferred to the LGP TPS for irradiation planning. For this study 8×10 inch EBT2 film (ISP Corporation, NJ) was used. EBT2 film has high spatial resolution, can be handled in room light, can be cut into any shape and size, and is tissue‐equivalent.^(43)^ Dose range of the EBT2 film is 0–10 Gy in red channel and greater than 10 Gy measurements can be done in green channel. (Further details about EBT2 film can be found in Richley et al. and Andres et al.)^44^, ^45^ For calibration, the EBT2 film was cut into small strips of size 3 cm x 2.5 cm and placed inside the Gamma Knife film holder tool without piercing the middle of the film. The strips were marked to indicate the film orientation. Film strips were exposed to different doses from 0.5 Gy to 10 Gy in the Gamma Knife Unit model B.

The same planning created for the gel measurement was used for the film measurement (see Table 1). The phantom was fixed in the frame so that the film insert was lying near the region where the planning X, Y, and Z coordinates matched within 0.5 mm positioning accuracy. Treatment was identical to the gel measurement, except for the modified prescription of 2 Gy to the 50% isodose level. The phantom along with the stereotactic frame was mounted on the LGK model B, and the plan was treated.

A flat bed scanner (Microtek 9800XL; Microtek Lab Inc., Santa Fe Springs, CA) was used to scan the exposed films in transmission mode. After a scanner warming time of 30 minutes, the calibration and measurement films were scanned five times to minimize the random noises and uncertainties that occurred during the scanning procedures. The film images were split into three color channels— red, green and blue. The pixel values obtained from each channel were converted into optical density and calibration curve of the 4th degree polynomial was determined for each channel using curve fitting function of ImageJ (National Institutes of Health, Bethesda, MD). Detailed film dosimetry procedure is described in Devic et al.^(46)^ We applied similar procedures for our study. The obtained dose map was compared with TPS calculated dose grid using in‐house generated MATLAB codes. Comparison tools used for 3D comparison were used for 2D comparison with film as well (See Materials & Methods Section E above).

## III. RESULTS

### A. Gel measurement results

Figure 3 shows the residual plot for the R2‐dose response of the MAGAT polymer gel dosimeter formulation four days postirradiation. R2 values ranged from 1.65 to $83\;s^{‐1}$ for doses from 0 to 17 Gy. A linear plot was made between R2 and dose.^(47)^ Appropriateness of the linear plot was assessed by examining the residual plots. Points in the plot were randomly dispersed around the horizontal axis, which indicates a reasonable fit to the data.

**Figure 4:**
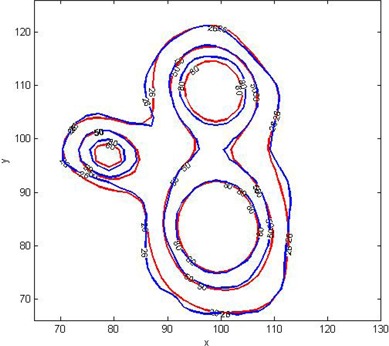
Dose distribution comparison in axial plane. Comparison of MAGAT polymer gel: MRI‐measured (blue line) and LGP‐calculated (red line) relative dose distributions of 26% (4.2 Gy), 50% (8 Gy), 80% (12.8 Gy), on an axial plane at Z=101 mm.

**Figure 5:**
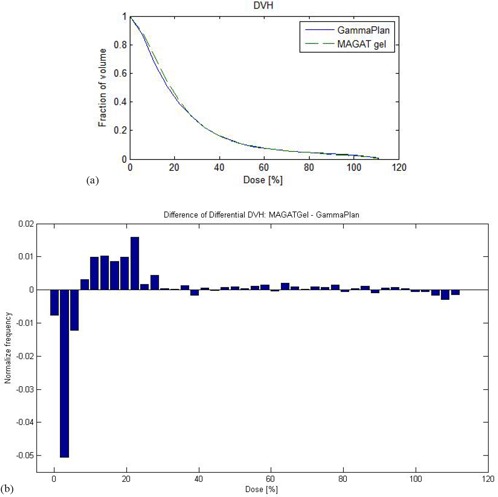
Dose‐volume comparisons (a) of measurements (green dotted line) and LGP calculations (blue solid line). Note that 100% in horizontal axis corresponds to 16 Gy. A diagram (b) showing the difference in differential dose‐volume histograms for measured and computed dose distributions. Relative number of voxels vs. dose at the voxel.

Figure 4 represent the measured and calculated dose distributions on axial plane at Z=101 mm. In this figure, 8 Gy (50%) and 12.8 Gy (80%) isodose lines agree within 1 mm distance and 2 mm distance, respectively, between measurements (blue line) and LGP calculations (red line).

(Figure 5a) shows the dose volume histogram (DVH) for calculated and measured dose data. Comparison shows close agreement between two methods. Minor deviation was observed between 4% (0.64 Gy) to 19% (3.04 Gy). This is clearly evident in (Fig. 5b), which is

the difference in differential dose‐volume histogram (DDDVH). It was obtained subtracting the number of voxels in dose bins for the measured dose from calculated dose. The DDDVH is normalized either by dividing by the total number of voxels or only the voxels expected to be in the bin. In this figure we have shown DDDVH for the whole 3D data comparison; hence, normalization was done by dividing with total number of voxels. A value of 0 for a bin means that both measured and calculated dose have the same number of voxels with a specific dose range in that bin. A bar or column height meeting at vertical y‐axis with a value of 0.01 means that measured and calculated doses have difference of 1% between them. (Figure 5b) shows that measured dose was greater than the calculated dose between 4% (0.64 Gy) to 19% (3.04 Gy). Beyond 19% (3.04 Gy) dose difference in the DDVH is negligible.

**Figure 6:**
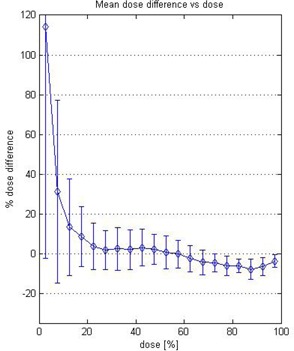
The dose‐dependent–dose‐difference diagram (D4 diagram) generated using percent difference ratio. The diamond symbols indicate the mean dose difference. The error bars are for one standard deviation. Note that the 100% corresponds to 16 Gy, which is the maximum dose in the calculation matrix.

The dose‐dependent–dose‐difference (D4 diagram) is shown in Fig. 6. The diamond symbols indicate the mean dose difference. The error bars are for one standard deviation. Note that the 100% corresponds to 16 Gy, which is the maximum dose in the calculation matrix. Figure 6 shows the mean dose difference between the measured and calculated values. Between 20% (3.2 Gy) to 65% (10.4 Gy), mean dose difference was around ±2%. Between 70% to 100%, mean dose difference was ±5%. For lower dose range below 3.2 Gy (20%), more than ±10% dose differences were observed.

**Figure 7:**
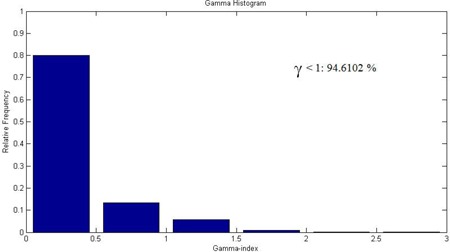
Histogram showing the gamma index distribution for MAGAT gel measurement. The criterion for the gamma index calculations was 2%/2 mm in axial plane. The calculation volume was limited to a subspace x=70 to 115, y=65 to 115, and z=90 to 114. More than 90% voxels included in the calculation had the value smaller than unity.

The gamma index histogram of LGP calculated vs. gel measured dose was calculated for (a) 3%/2 mm, (b) 3%/1 mm, and (c) 2%/2 mm tolerance criteria in axial plane. Figure 7 shows the gamma index histogram for 2%/2 mm tolerance criteria in axial plane. The gamma evaluation showed that gel measured dose satisfied all the three tolerance criteria with high pass rate. More than 94% voxels included in the calculation had the value smaller than unity. Summary of the gamma evaluation pass rates for gel measurement is given in Table 2.

**Table 2:** Pass rates of gamma evaluation using five tolerance criteria (3%/2 mm, 3%/1 mm, 2%/2 mm, 1%/1 mm, and 0.5%/0.5 mm; units, %).

	3%/2 mm	3%/1 mm	2%/2 mm	1%/1 mm	0.5%/0.5 mm
Film vs. LGP	97	95	96	86	80
Gel vs. LGP	97	94	95	84	78

(Figures 8a)and (8b) show the isodose overlay and gamma index map for 2%/2 mm tolerance criteria in the axial plane between gel measured (dotted line) and TPS calculated (solid line) dose at Z=101 mm. Comparison showed low gamma index values in the irradiated areas around the shot centers. High gamma index values (>1) were observed in the far periphery areas of the gel phantom. Figure 2 represents the actual radiation exposure region in the EBT2 film and MAGAT gel dosimeter using treatment parameters created in LGP given in Table 1. Small circles in the gel image shown in (Fig. 2b) represent the calibration vials which were scanned together with the spherical glass phantom (big black circle in the image). Film measurement results are discussed in the following section.

**Figure 8:**
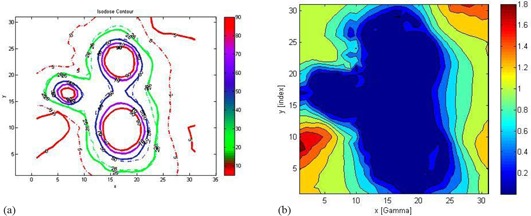
Isodose overlay comparison of gel measured (dotted line) and computed dose distributions (solid line) on axial plane at Z=101 mm. Isodose lines (a) for 5% (0.8 Gy), 26% (4.2 Gy), 50% (8 Gy) 70% (11.2 Gy), and 90% (14.4 Gy) of measurements and calculations are plotted. The units of x‐ and y‐axes are in mm. Gamma index map (b) for 2%/2 mm tolerance criteria in the axial plane. Comparison shows low gamma index values in the irradiated areas around the shot centers.

### B. Film measurement results

**Figure 9:**
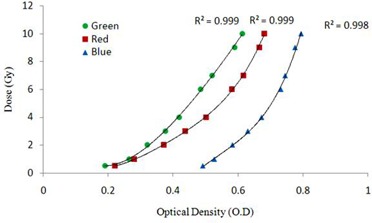
Optical density vs. absolute dose curves of EBT2 film in the 0–10 Gy dose range for green, red, and blue channels.

**Figure 10:**
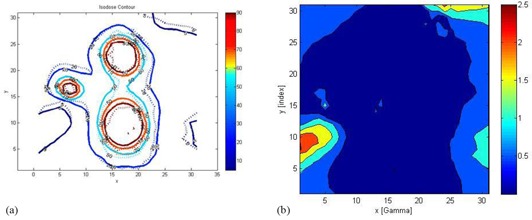
Isodose overlay comparison of film‐measured (dotted line) and computed dose distributions (solid line) on transverse plane. Isodose lines (a) for 5% (0.2 Gy), 26% (1.04 Gy), 50% (2 Gy), and 70% (2.8 Gy) and 90% (3.6 Gy) of measurements and calculations are plotted. The units of x‐ and y‐axes are in mm. Gamma index (b) map for 2%/2 mm tolerance criteria in the axial plane.

Figure 9 shows the fourth degree polynomial fit between optical density and dose. The red and green channels had an $R^{2}$ value of 0.9999 in 0–10 Gy range. The pixel data of red channel was used for further analyses of measurement films, as the correlation curve of the red channel showed highest response gradient in comparison with other channels.^(48)^ Comparison of film measured and LGP calculated dose distribution were done in the axial plane at Z=101 mm, represented in (Fig. 10a). Isodose lines 5% (0.8 Gy), 26% (1.04 Gy), 50% (2 Gy), 70% (2.8 Gy), and 90% (3.6 Gy) were plotted. The relative dose comparison showed close agreement within 1 mm distance between the film‐measured dose and calculated dose. For the gamma index analysis, a region around the three shots positioned between X=70 mm to 115 mm, Y=65 mm to 125 mm, and Z=101 mm was chosen (see (Fig. 10a).

In (Fig. 10b), gamma index map for 2%/2 mm tolerance criteria is plotted on the axial plane. Comparison showed low gamma index values in the irradiated areas around the shot's center. High gamma index values (>1.5) were observed in the periphery areas of the EBT2 film.

**Figure 11:**
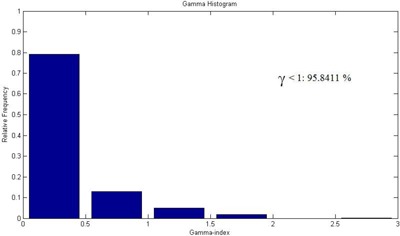
Histogram showing the gamma index distribution for EBT2 film measurement. The criterion for the gamma index calculations was 2%/2 mm in axial plane. The calculation region was limited to a subspace x=70 to 115, y=65 to 115, and z=101.

The gamma index histogram of LGP calculated vs. film‐measured dose was calculated for (a) 3%/2 mm, (b) 3%/1 mm, and (c) 2%/2 mm tolerance criteria in axial plane. Figure 11 shows the gamma index histogram of LGP calculated vs. film‐measured dose for 2%/2 mm tolerance criteria in axial plane. The gamma evaluation showed that film measured dose satisfied all the three tolerance criteria with high pass rate. More than 95% voxels included in the calculation had the value smaller than unity. Summary of the gamma evaluation pass rates for film measurement is given in Table 2.

Gamma evaluation with gel and film measurement showed that the test plans satisfied 3 mm/2%, 3 mm/1%, 2 mm/2% with >96%, >94%, and <95% pass rates, respectively. For tolerance criteria of 1 mm/1% and 0.5 mm/0.5%, gel measurement pass rates were 84% and 78%, respectively, whereas film measurement pass rates were 86% and 80% for each criteria (see Table 2).

**Figure 12:**
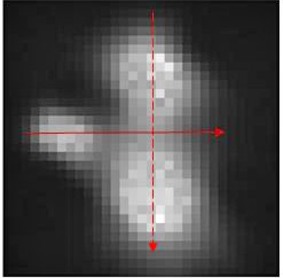
The arrows indicating the direction in which the horizontal (smooth line) and vertical profile (dashed line) were obtained for comparison between Leksell GammaPlan (LGP), gel dosimeter, and EBT2 film.

**Figure 13:**
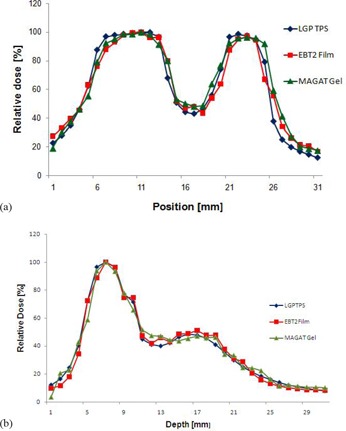
Comparison of experimentally obtained dose distributions represented by dose profiles in axial images (profiles were taken in the direction as shown in (a) representing the vertical profile between LGP calculated, film measured and gel measured doses; (b) representing horizontal profile between LGP‐calculated, film‐measured, and gel‐measured dose.

Figure 12 shows the direction in which horizontal (smooth line) and vertical profile (dashed line) were obtained for comparison between calculated and measured doses. (Figures 13a)and (13b) show the line profile comparison between LGP calculated, gel‐measured and film‐measured dose in vertical and horizontal directions. These values agreed very closely without any distinct mismatched regions. Note that the prescription dose for gel measurement was 16 Gy maximum dose and for film measurement was 4 Gy maximum dose. Film profiles were rescaled to match with the profiles of gel measurement.

## IV. DISCUSSION

### A. Outcome of 3D and 2D measurements on plugging

In the planning after shielding placement to the Y‐shaped target, the isodoses were considerably modified and the main effect was the reduction in dose to the critical structures (OARs). This is clearly evident through the 26% (4.16 Gy) isodose line which initially was lying on the OARs. After applying plugs, 26% isodose line is moved away from the OAR in the left side (pink color) and runs along the edge of the OAR in the right side (orange color) (see (Fig. 1b). This effect is exactly what we verified using 3D and 2D measurements.

In this work, the use of gamma evaluation method was demonstrated to verify the delivery accuracy of the procedure for the whole 3D data and 2D data. Gamma index pass rates went from 97% at (3%/2 mm) to 78% at (0.5%/0.5 mm). As the tolerance criteria became stricter (i.e., 1%/1 mm and 0.5%/0.5 mm), pass rates became lower. Higher gamma index values (>1.6) were located in the far periphery area in both film and gel measurement. This was more pronounced in the gel measurement due to steep response to irradiation doses in the low dose range (see (Fig. 8a)and (8b). Note that the film measurement is restricted to planar measurement, whereas gel measurement is a volumetric measurement. The relative number of measurement points in the periphery area is greater in gel volumetric measurement compared to film planar measurement. The voxel size for LGP dose calculations was $2.5\;\times \;2.5\;\times \;2.5\;{\text{mm}}^{3}$, while the voxel size was $0.5\times 0.5\times 2\;{\text{mm}}^{3}$ for the gel dose measurements. The calculated and measured 3D dose distributions were compared by interpolating them in a common space with $1.0\times 1.0\times 1.0\;{\text{mm}}^{3}$ grids. The linear interpolation in 3D was performed using a numerical routine of MATLAB. Our future studies will focus on imaging the gel container with 1 mm slice thickness that would meet strict tolerance criteria such as 1 mm/1% and 0.5 mm/0.5% with higher pass rate.

Overall in this study, isodose overlay, gamma evaluation for (a) TPS versus film and (b) TPS versus gel measurement and line profile measurements showed close agreement. DVH analysis was also performed to examine the difference between TPS dose and gel measured dose. At present, we could not compare the DVH specific to the structures because the current version of our in‐house software does not use the spatial location data of the structures for the DVH analysis.

### B. Role of gel dosimetry in future versions of LGP

Recent developments in LGP TPS (LGP version 10.1) include the incorporation of tissue heterogeneity correction in calculation algorithm. This new feature overcomes the limitation of older LGP versions, which lack the ability to predict the rapid dose fall off due to air cavities behind or near lesion that leads to overestimation of dose. Such overestimation was demonstrated previously through gel measurement by calculating 3D dose distribution in inhomogeneous phantom.^(3)^ Dose maps in the inhomogeneous phantom were found to be spatially different. As gel was already used successfully in that study for measuring 3D dose distribution near the area of tissue heterogeneities, there is no doubt that it has a major role in predicting dose distributions for calculations based on recent versions of LGP which take heterogeneity corrections into account. In future, a patient treatment plan designed in LGP with both heterogeneity correction and blocking will be even more complex and may require more verification. The 3D and 2D evaluation methods which we have shown in this study would be useful as QA method for comparison of dose distributions in GK radiosurgery, not only for plug planning but for any complex situations involving steep dose gradients.

## V. CONCLUSIONS

In numerous clinical situations, the source plugging procedure is chosen for shielding the critical organs while making a treatment plan in Gamma Knife SRS. By performing verification through gel dosimetry, we could directly visualize what a shielding procedure really does to the outcome of the resultant planning. We have demonstrated a method to verify in 3D a treatment based on source plugging or blocking using gel dosimetry. In addition to 3D verification, we also have demonstrated a method for film‐based treatment verification for Gamma Knife plans, especially for source plugging to validate the TPS calculations.

All the physical parameters used in this study for comparison exhibited a small difference between measured dose and TPS calculations. Gamma index evaluation for verification confirmed our results with high pass rates.

In summary, high gradient regions in the Gamma Knife plan are quite important and do have the highest uncertainty. Performing QA for such situations is important for better informing the neurologist of uncertainty in these regions, and potentially improving dose calculation algorithms. Through this work we validated delivery of a LGK plan with plugged fields using gel dosimetry. Based on our study and results, we propose gel dosimeter as a QA test tool for Gamma Knife users.

## ACKNOWLEDGMENTS

We wish to gratefully acknowledge the support of AERB, India for this work through research project no N‐964. We also would like to thank technical staff Mr. Kripal and Mr. Hargovind Jeena for their help during the imaging procedures.
